# PP109 Which Review Is Right For You? Choosing A Review Methodology

**DOI:** 10.1017/S0266462324002708

**Published:** 2025-01-07

**Authors:** Mary Edwards, Lavinia Ferrante di Ruffano

## Abstract

**Introduction:**

While systematic reviews (SRs) are regarded as the gold standard in healthcare evidence reviewing (and a requirement of many health technology assessments [(HTAs)]), other types of review also play an important role throughout a product’s lifecycle. Drawing on more than thirty years’ experience in conducting reviews, we present key points to consider when deciding which review type might be required.

**Methods:**

SRs are recommended when a comprehensive search and synthesis approach is required, for example HTAs. They have highly structured methods, emphasizing bias minimization, transparency, and replicability. “Rapid,” “pragmatic,” or “targeted” reviews are increasingly popular due to their accelerated timelines and reduced costs, with methodological shortcuts possible at various stages. Scoping reviews explore what is known about a topic and typically have a broad research question. “Reviews of reviews” or “overviews” identify existing SRs on an established topic. Finally, “living reviews” follow the same process as an SR or rapid review but incorporate new evidence on a continual or regular basis.

**Results:**

Rapid reviews may be appropriate when flexibility exists regarding the scope and review methods. Any limitations due to methodological shortcuts must be acknowledged in a transparent manner. Scoping reviews are useful for pioneering research ahead of an SR, or early in a product’s development phase, when an overall understanding of the evidence base is required. Reviews of reviews are particularly useful when the size of the primary study literature means that a review of primary studies would be unfeasible. Living reviews are best suited to topics where the evidence base is changing rapidly, or the best information is needed quickly.

**Conclusions:**

When considering conducting or commissioning a review, organizations should consider the intended audience for the review, the resources, time, and budget available, and the size of the existing literature. Although SRs remain the gold standard, a rapid review, scoping review, or review of reviews may offer a more suitable way to approach a given research question.

